# Impact of rapid maxillary expansion on nasal microbiota in mouth-breathing children: a prospective cohort study

**DOI:** 10.1186/s12903-026-08148-2

**Published:** 2026-03-28

**Authors:** Ruoan Chen, Haiyi Wang, Shiying Hu, Jianfeng Wang, Limin Wei

**Affiliations:** 1 School and Hospital of Stomatology, Wenzhou Medical University, Wenzhou, China; 2 Orthodontic Department, School and Hospital of Stomatology, Wenzhou Medical University, Wenzhou, China; 3 Pediatric Dentistry Department, School and Hospital of Stomatology, Wenzhou Medical University, Wenzhou, China

**Keywords:** Rapid maxillary expansion (RME), Mouth breathing, Nasal microbiota, 16S rRNA gene

## Abstract

**Background:**

Maxillary transverse deficiency (MTD) is a common malocclusion in children and is often associated with mouth breathing. Rapid maxillary expansion (RME) is a conventional treatment for MTD; however, its association with nasal microbiota composition in mouth-breathing children remains unclear. This study aimed to evaluate nasal microbial community composition before and after RME.

**Methods:**

This was a prospective cohort study. Thirty-two children aged 5–14 years were enrolled, including 16 with maxillary transverse deficiency (8 mouth-breathing and 8 nasal-breathing) and 16 healthy controls. Nasal swabs were collected before and one month after RME in the MTD groups and once in the control group. Microbial DNA was sequenced using the Illumina MiSeq platform. Analyses included alpha diversity, taxonomic composition, and differential abundance across groups.

**Results:**

No significant differences in alpha diversity were observed between mouth-breathing children and controls, or before and after RME. *Firmicutes, Proteobacteria, and Actinobacteria* were dominant across groups, and compositional differences were observed after RME. In the mouth-breathing subgroup, the relative abundance of *Streptococcus* significantly decreased after RME (P < 0.05), while the relative abundance of *Actinobacteria* increased. Overall, post-RME samples in the mouth-breathing subgroup showed compositional shifts, including a trend toward a profile more similar to that observed in healthy controls.

**Conclusions:**

RME was associated with short-term changes in nasal microbiota composition while overall alpha diversity remained relatively stable. In mouth-breathing children, post-RME compositional shifts included reduced Streptococcus relative abundance. These findings should be interpreted as exploratory associations, and further studies with longer follow-up, standardized breathing-pattern reassessment, and clinical/inflammatory outcome measures are needed to clarify their clinical significance.

**Trial registration:**

Chinese Clinical Trial Registry (ChiCTR), ChiCTR2500112875, registered on November 20, 2025. Retrospectively registered. Available at: https://www.chictr.org.cn/showproj.html?proj=290888.

**Supplementary Information:**

The online version contains supplementary material available at 10.1186/s12903-026-08148-2.

## Introduction

Maxillary transverse deficiency (MTD) is a common malocclusion in children, characterized by inadequate maxillary growth in the transverse dimension, resulting in dental arch constriction, posterior crossbite, crowding, and high palatal vaults [1]. MTD has also been associated with reduced nasal and pharyngeal airway dimensions and altered breathing patterns [2]. However, mouth breathing in children may arise from multiple etiologies (e.g., allergic rhinitis, adenoidal/tonsillar hypertrophy, and other upper airway conditions), and its relationship with craniofacial development is likely multifactorial and potentially bidirectional.

Rapid maxillary expansion (RME) is a conventional intervention for MTD. By applying orthopedic force to the midpalatal suture, RME induces skeletal remodeling and enlarges the maxillary arch [3, 4]. Previous studies have reported that RME may be associated with changes in tongue posture, nasal cavity dimensions, and airway-related parameters [5, 6]. These structural changes may influence nasal airflow and airway resistance and may be relevant to breathing patterns in some children. Given that the maxilla forms a major part of the nasal framework, RME may also affect the nasal microenvironment.

The nasal cavity harbors a distinct microbial community dominated by aerobic and facultative anaerobic bacteria, while the oral microbiota is largely anaerobic [7]. Although both sites are anatomically connected, they usually maintain partially distinct ecological niches. Disruption of this balance has been associated with respiratory conditions such as chronic rhinosinusitis and nasopharyngeal carcinoma [7, 8]. Previous orthodontic studies have primarily focused on oral microbiota [9–11], whereas the potential association between orthodontic treatment and nasal microbial ecology remains largely unexplored.

Because RME may alter craniofacial and nasal structural characteristics, it is plausible that it may also be associated with changes in nasal microbiota composition. Therefore, this prospective cohort study used 16S rRNA gene high-throughput sequencing to characterize nasal microbial community composition in children with MTD and to evaluate changes before and after RME, with subgroup analyses according to breathing pattern.

## Methods

### Study design and participants

This prospective cohort study was conducted at the Affiliated Stomatology Hospital of Wenzhou Medical University between October 2023 and July 2024. A total of 32 children aged 5–14 years (14 boys and 18 girls; mean age 7.9 years) were enrolled. Sixteen children were diagnosed with maxillary transverse deficiency (MTD), including 8 classified as mouth-breathing and 8 classified as nasal-breathing. Sixteen age-matched healthy children with normal maxillary transverse development served as controls. The MTD nasal-breathing subgroup was included to help distinguish changes potentially related to RME from those associated with breathing pattern.

For the MTD group, inclusion criteria were transverse maxillary deficiency requiring rapid maxillary expansion (RME), based on routine orthodontic assessment and treatment planning. Clinical examination could reveal suggestive findings such as apparent transverse insufficiency, maxillary arch narrowing, or dental crowding; however, these findings alone were not considered sufficient for definitive diagnosis. Posterior crossbite on clinical examination, when present, was regarded as an important clinical sign strongly suggestive of MTD and the possible need for expansion. All children in the MTD group underwent CBCT as part of routine orthodontic evaluation, and CBCT-based transverse assessment, including maxillary and mandibular basal bone width evaluation using the Penn analysis framework, was used to support MTD diagnosis and treatment planning by the same senior orthodontist. Additional orthodontic records, including routine clinical examination, dental casts, and occlusal photographs, were also reviewed.

Breathing-pattern subgrouping was performed before treatment using a combination of medical record review, caregiver report, and clinical observation. For the mouth-breathing subgroup, supporting information included medical record documentation of “mouth breathing” or “chronic mouth breathing”, history records suggestive of chronic mouth breathing (e.g., frequent/long-term snoring, nasal obstruction symptoms, nighttime mouth opening), caregiver-reported nighttime mouth breathing/snoring, and clinical signs (e.g., habitual open-mouth posture, lip eversion/lip incompetence, mouth-breathing facial features, and chairside clinical observation of oral airflow, including a fogging mirror-based assessment). Children in the nasal-breathing subgroup had no clinically observed mouth-breathing symptoms, and caregivers reported no mouth breathing during sleep.

Control participants had normal maxillary transverse development, no anterior or posterior crossbite, no maxillary arch constriction, and no orthodontic treatment need. Exclusion criteria for both groups included use of antibiotics, probiotics, or nasal sprays within the previous month; history of adenoidectomy, tonsillectomy, or other nasal/nasopharyngeal surgery; prior airway-related treatment (e.g., continuous positive airway pressure [CPAP], oral appliances, or uvulopalatopharyngoplasty [UPPP]); and syndromes, chronic systemic disease, craniofacial deformities, complete nasal obstruction, septal perforation, or any known acute disease of the nasal cavity, oral cavity, paranasal sinuses, salivary glands, or pharynx. Children with prior adenotonsillar surgery were excluded because such procedures may affect nasal microbial community composition. The study protocol was approved by the Ethics Committee of the Affiliated Stomatology Hospital of Wenzhou Medical University (Approval No. WYKQ2024003). Additional details on orthodontic assessment and breathing-pattern subgrouping are provided in the Supplementary Methods and Supplementary Table S1, and a participant flow diagram is provided in Supplementary Figure S6.

### Sample collection

**Fig. 1:**
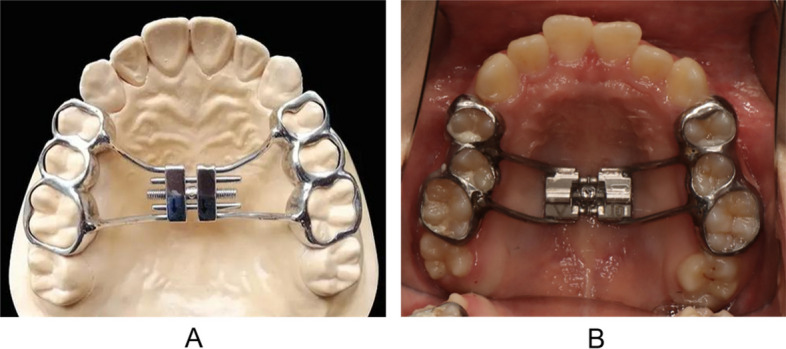
Hyrax expander. **A** Schematic model of the Hyrax expander; **B** Intraoral view of the Hyrax expander

All 16 MTD patients underwent RME using a fixed Hyrax expander (Fig. 1). Activation started 48 h after bonding, with two quarter-turns daily (0.25 mm per turn) for 2–3 weeks, until the maxillary arch width was coordinated with the mandibular arch. Retention was maintained for 3–6 months. At the post-RME follow-up visit, a symptom-focused chairside reassessment (caregiver report and clinical observation, including a fogging mirror–based assessment of oral airflow) was performed as part of routine follow-up; however, standardized objective otolaryngological reassessment was not conducted.

**Fig. 2:**
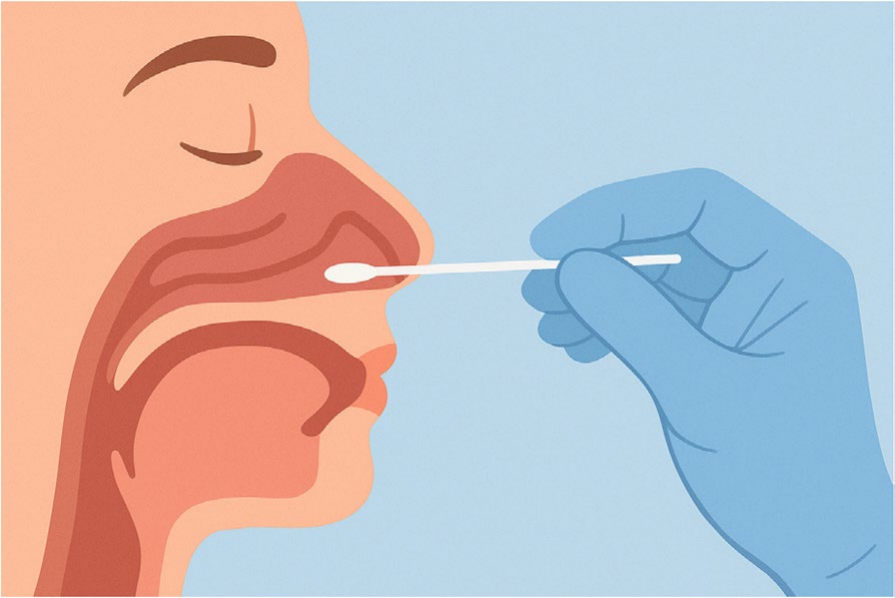
Schematic diagram of nasal swab sampling

Nasal swab samples were collected using sterile flocked swabs according to standard protocols (Fig. 2) [12, 13]. For MTD patients, samples were collected at baseline and approximately one month after completion of active expansion (during the retention phase). This post-expansion time point was selected to evaluate early (short-term) microbiota compositional changes after RME. Controls were sampled once. This yielded five groups, as shown in \* MERGEFORMAT Table 1: control (C, n = 16), mouth-breathing pre-RME (M, n = 8), nasal-breathing pre-RME (N, n = 8), mouth-breathing post-RME (MH, n = 8), and nasal-breathing post-RME (NH, n = 8), for a total of 48 samples. Swabs were immediately placed in sterile tubes, stored at − 80 °C, and processed within six months.

**Table 1:** Sample grouping

Group	Sample type	Sample size
C	control	16
M	mouth-breathing pre-RME	8
N	nasal-breathing pre-RME	8
MH	mouth-breathing post-RME	8
NH	nasal-breathing post-RME	8

### DNA extraction, PCR amplification, and sequencing

Total microbial DNA was extracted from nasal swabs using the FastPure Soil DNA Isolation Kit (MJYH, Shanghai, China; catalog no. T09-96) according to the manufacturer’s instructions. DNA concentration and purity were assessed using a NanoDrop spectrophotometer, and DNA integrity was evaluated by agarose gel electrophoresis.

The V3–V4 region of the 16S rRNA gene was amplified using primers 338 F (5′-ACTCCTACGGGAGGCAGCAG-3′) and 806R (5′-GGACTACHVGGGTWTCTAAT-3′) [13]. Polymerase chain reaction (PCR) reactions were performed in triplicate for each sample, and pooled amplicons were purified and quantified. Amplicon sequencing libraries were constructed using the NEXTFLEX Rapid DNA-Seq Kit according to the manufacturer’s instructions, and paired-end sequencing (2 × 300 bp, PE300) was performed on the Illumina MiSeq platform. The quality of DNA and PCR products was verified by agarose gel electrophoresis. Full uncropped PCR gel images are provided in Supplementary Figures S3–S5; representative DNA electrophoresis results are shown in Supplementary Figures S1–S2.

### Bioinformatics and statistical analysis

Raw sequencing reads were processed with fastp [14] for quality filtering and FLASH [15] for paired-end merging. Operational taxonomic units (OTUs) were clustered at 97% similarity using USEARCH [16, 17], and taxonomic assignment was conducted with the RDP classifier [18] at a confidence threshold of 70%. Functional prediction was performed using PICRUSt2 [19].

Alpha diversity indices, including the Chao1 richness estimator (Chao1) and the Shannon diversity index (Shannon), were calculated with mothur [20], and rarefaction curves were generated using QIIME [21]. Group differences in alpha diversity were assessed with the Wilcoxon rank-sum test. Beta diversity was evaluated by principal coordinate analysis (PCoA) and principal component analysis (PCA) based on unweighted UniFrac distances, with statistical testing via permutational multivariate analysis of variance (PERMANOVA). Taxonomic composition was visualized at the phylum and genus levels using R software. Differential taxa across groups were identified with the Kruskal–Wallis H test.

## Results

### Taxonomic annotation and clustering

**Fig. 3:**
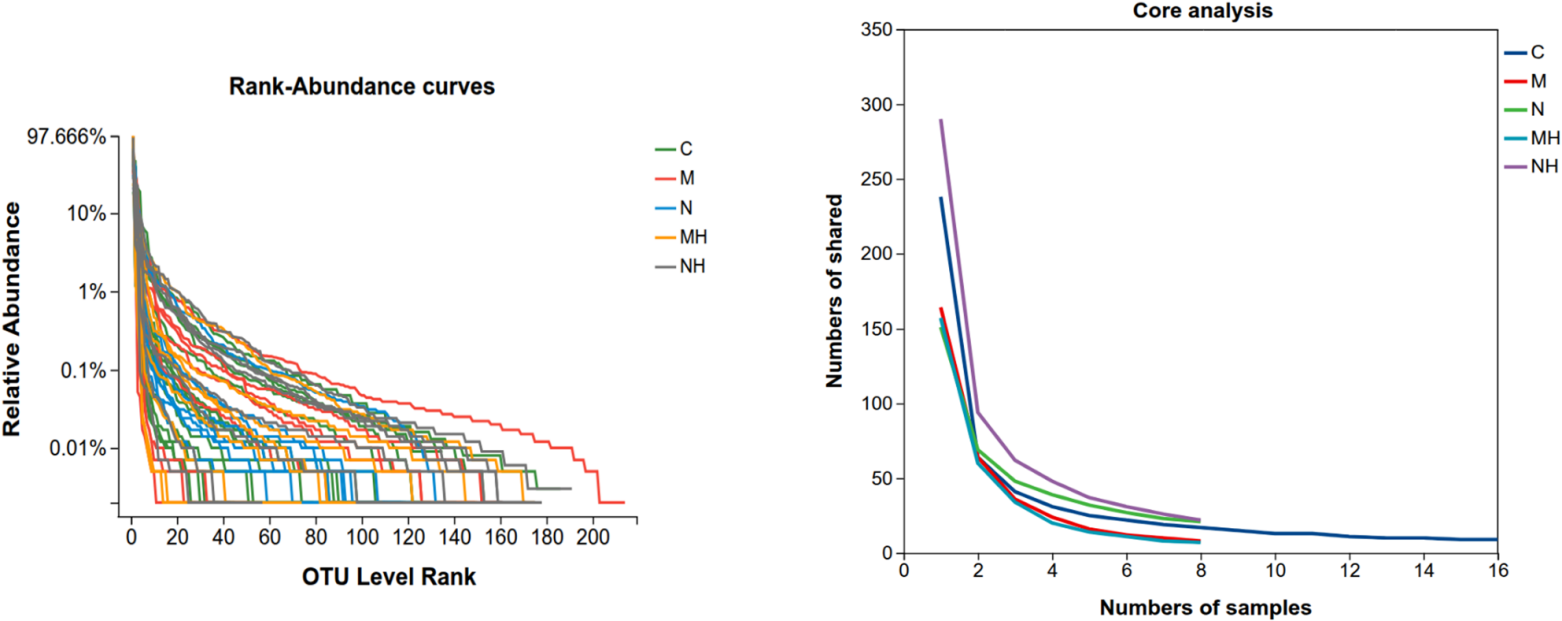
**A** Rank-abundance curves: Each curve represents one nasal microbiota sample, with different colors indicating different groups. **B** Core analysis: Each curve represents one nasal microbiota sample, with different colors indicating different groups

A total of 2,988,701 high-quality sequences were obtained, with an average read length of 423 bp. At a 97% similarity threshold, 2,702 OTUs were clustered, spanning 41 phyla, 116 classes, 271 orders, 46 families, and 941 genera. Rank-abundance curves showed a steep decline, indicating an uneven distribution dominated by a few abundant taxa, while core species curves reached a plateau, confirming sufficient sequencing depth (Fig. 3).

### Alpha diversity

**Fig. 4:**
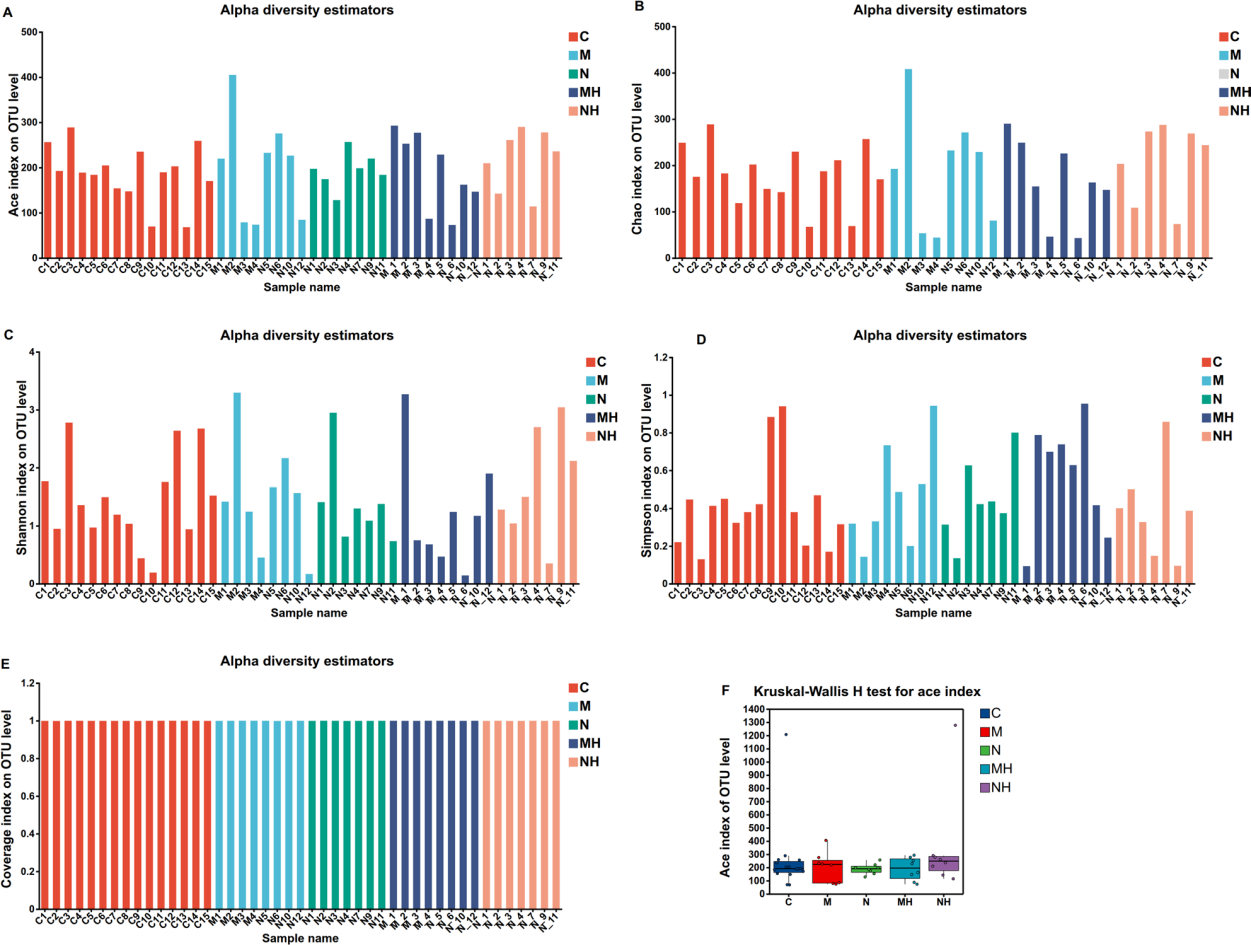
**A** ACE index comparison, reflecting community richness. **B** Chao1 index comparison, reflecting community richness. **C** Shannon index comparison, reflecting community diversity. **D** Simpson index comparison, reflecting community diversity. **E** Coverage index comparison, reflecting sequencing coverage. **F** Statistical comparison of Chao1 index among groups

**Fig. 5:**
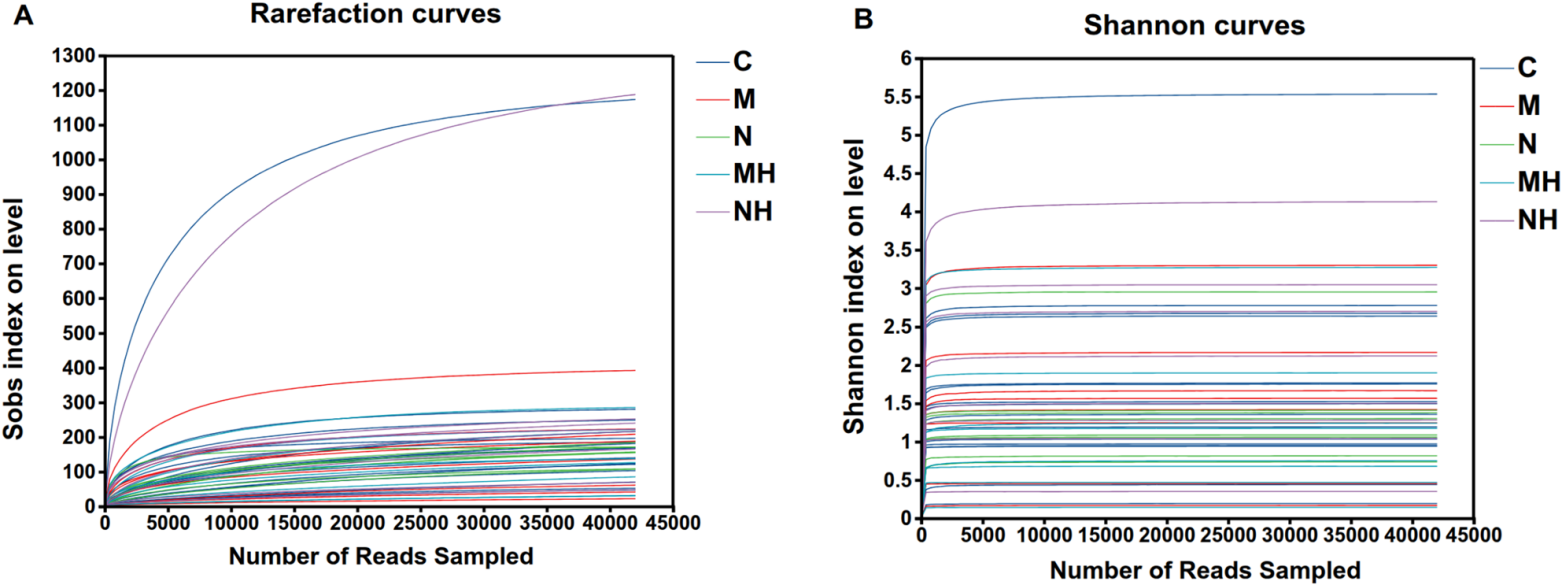
**A** Sobs rarefaction curves, **B** Shannon rarefaction curves. Each curve represents an individual sample, with different colors denoting different groups

Alpha diversity indices demonstrated generally high microbial richness and diversity. Chao1 and Abundance-based Coverage Estimator (ACE) indices were consistently > 1, Shannon indices mostly > 1, Simpson Diversity Index (Simpson) indices < 0.6, and Good’s coverage (Coverage) indices > 0.99, indicating adequate sampling depth (Fig. 4A–E). However, no significant differences in alpha diversity were observed between MTD and control groups, nor before and after RME in either subgroup (Fig. 4F, P > 0.05). Rarefaction curves for both observed species (Sobs) and Shannon indices approached saturation, further supporting adequate sequencing depth (Fig. 5).

### Beta diversity

**Fig. 6:**
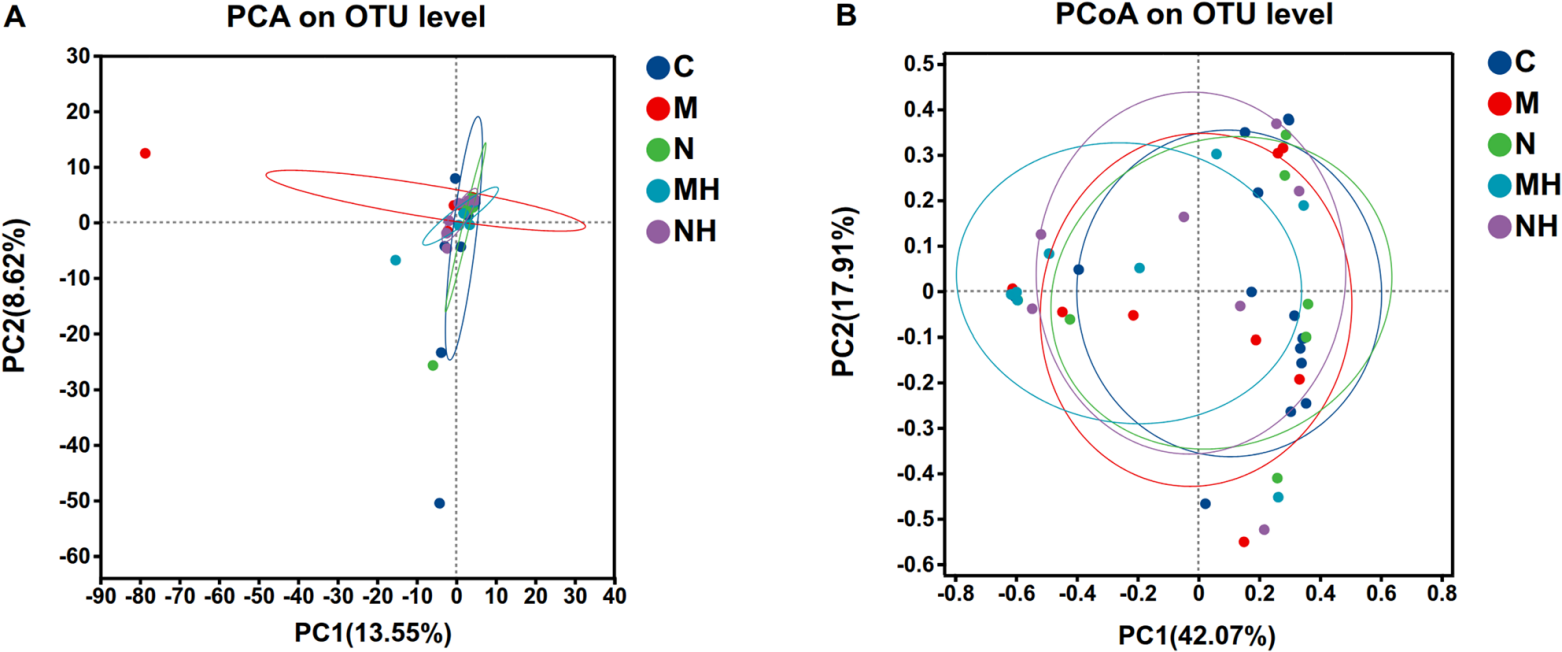
**A** Principal Component Analysis (PCA). The X- and Y-axes represent two selected principal components, with percentages indicating the proportion of variance explained. Each point corresponds to a sample, and different colors or shapes represent different groups. The closer the points, the more similar their microbial compositions. **B** Principal Coordinates Analysis (PCoA). Each point represents one sample, and ellipses denote clusters of samples from the same group. The closer the points, the more similar the microbial communities. Smaller overlaps between ellipses indicate clearer separation and greater differences in community structure among groups

**Fig. 7:**
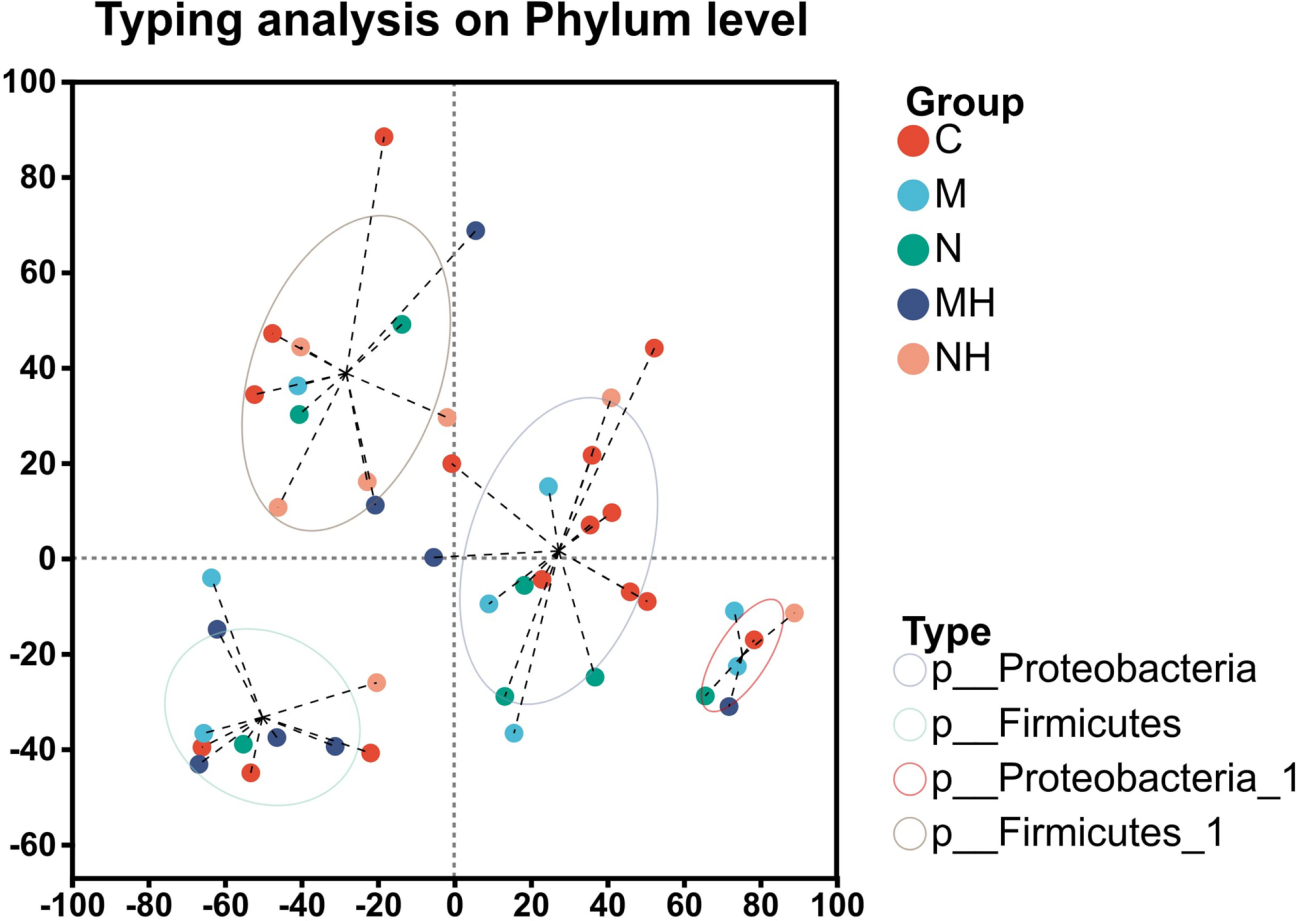
Each point represents an individual sample, with shape and color indicating group identity. The enclosed areas denote the 95% confidence intervals. Samples clustered into four distinct groups

Principal component analysis (PCA) and principal coordinates analysis (PCoA) analyses revealed broadly similar microbial community structures across groups, though intra-group variability was relatively high (Fig. 6). Clustering analysis divided samples into four enterotypes dominated by *Proteobacteria* and *Firmicutes* (Fig. 7).

### Community composition

**Fig. 8:**
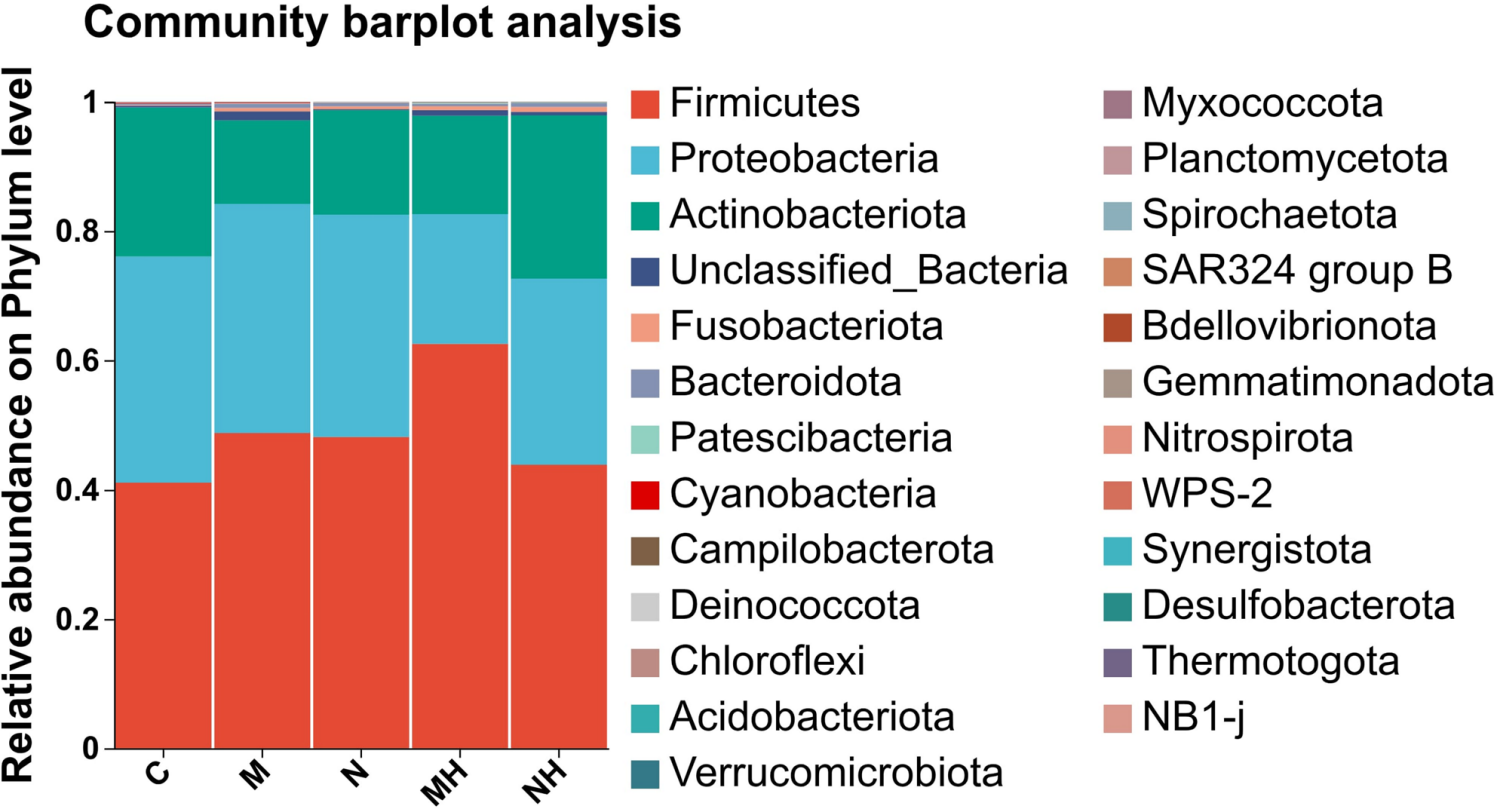
Bar plot of community composition at the phylum level. Bar plots are arranged in descending order of relative abundance. Each color represents a distinct taxon, with block size indicating relative abundance. The right panel shows the corresponding taxon names

At the phylum level, *Firmicutes*, *Proteobacteria,* and *Actinobacteria* were consistently dominant across all groups, though their relative abundances varied. In mouth-breathing children, post-RME samples showed higher relative abundance of Firmicutes and Actinobacteria and lower relative abundance of Proteobacteria compared with pre-RME samples. In nasal-breathing children, post-RME samples showed a slight increase in Actinobacteria and decreases in Firmicutes and Proteobacteria (Fig. 8).

**Fig. 9:**
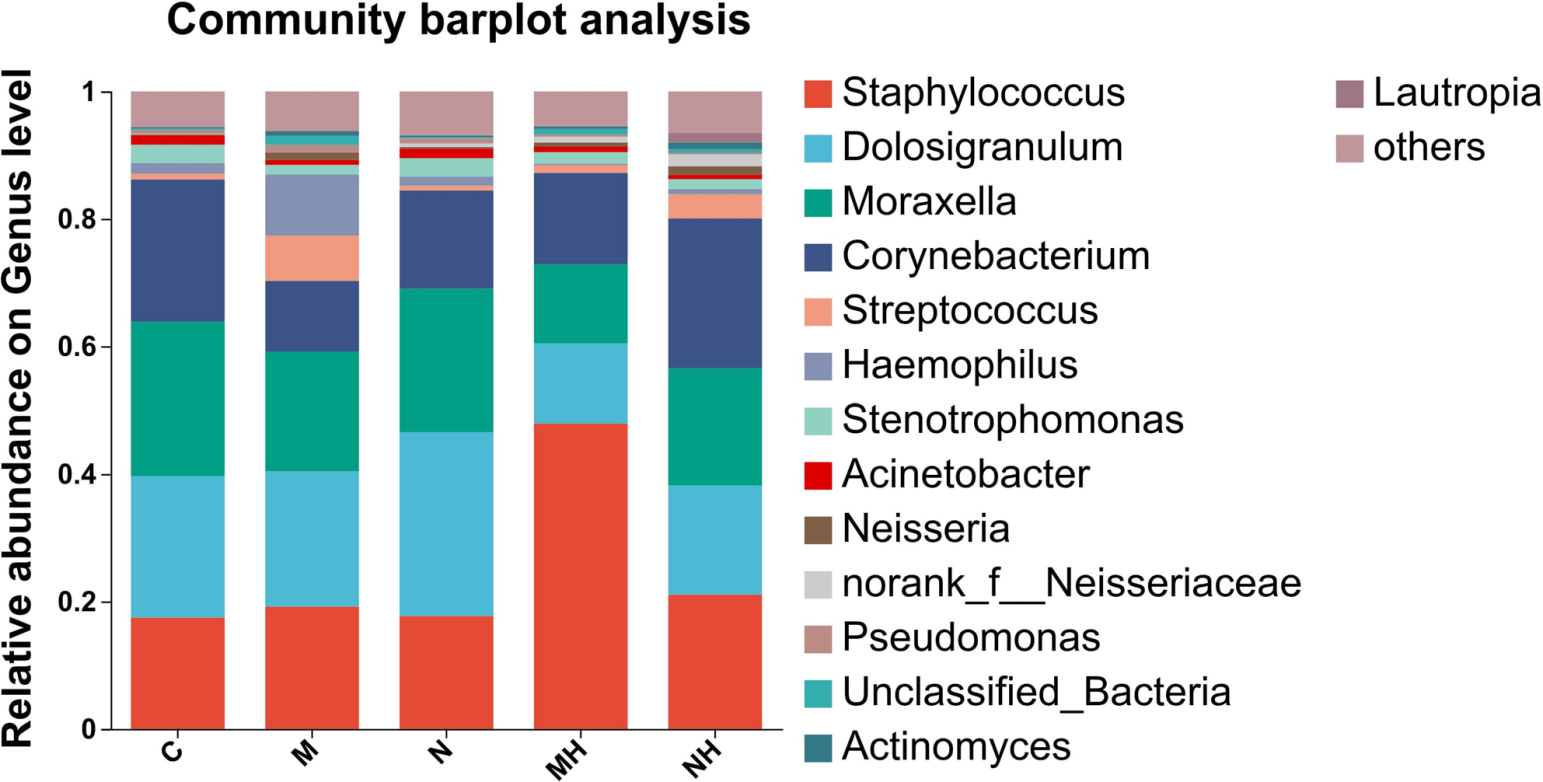
Bar plot of community composition at the genus level. Bars are arranged in descending order of relative abundance. Each color represents a different genus, and the size of each color block indicates its proportional abundance. The corresponding genera are shown on the right

**Fig. 10:**
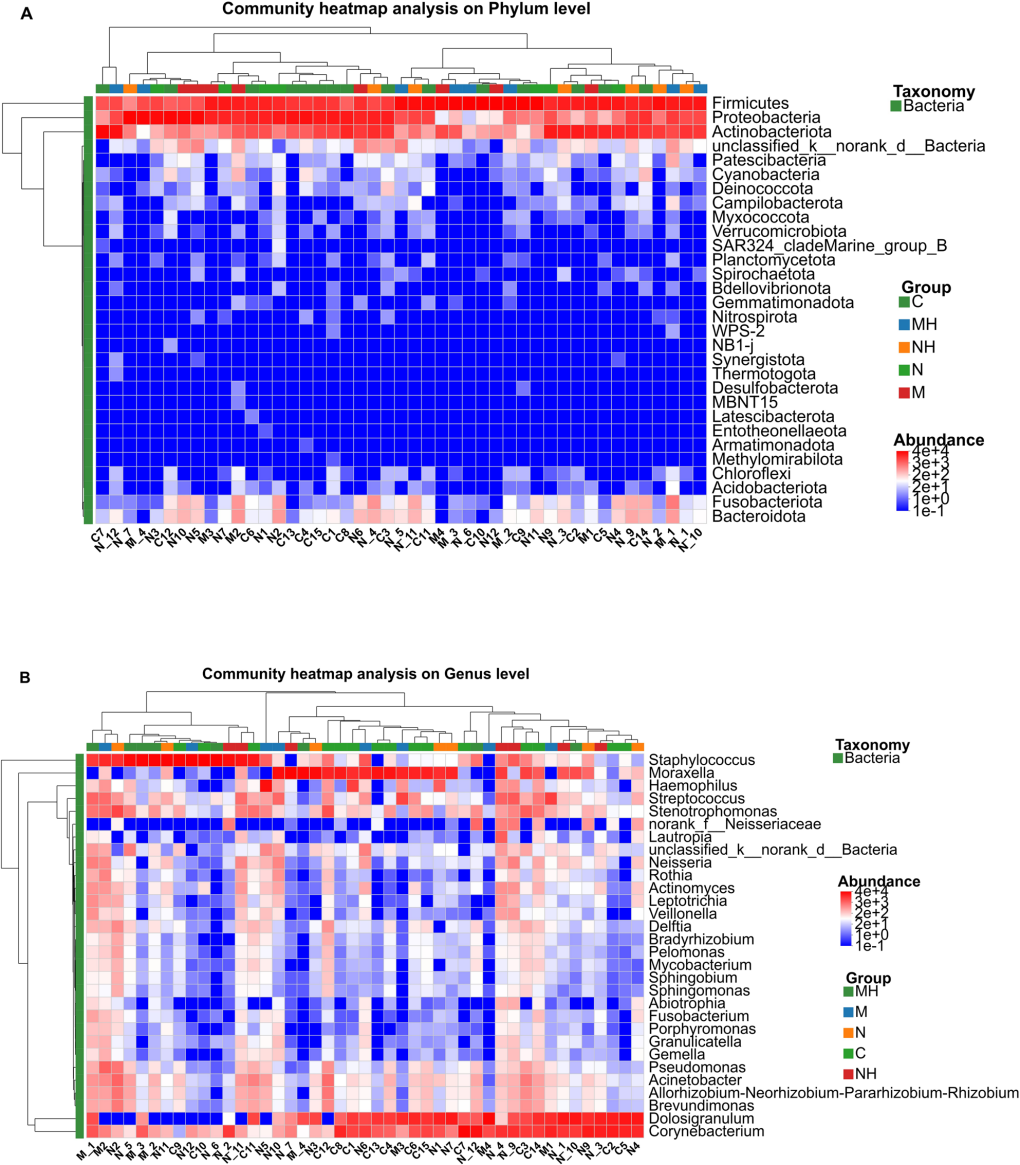
**A** Heatmap of community composition at the phylum level. **B** Heatmap of community composition at the genus level. The figure displays the relative abundance of the 30 most prevalent bacterial taxa. The horizontal axis represents samples, and the vertical axis represents taxon names

At the genus level, the leading taxa included *Moraxella, Corynebacterium, Dolosigranulum,* and *Staphylococcus,* though their proportions differed among groups. After RME, *Staphylococcus* and *Corynebacterium* increased, particularly in the mouth-breathing subgroup, while *Moraxella, Dolosigranulum,* and *Haemophilus* declined (Fig. 9). Heatmaps highlighted similar compositional shifts at both phylum and genus levels (Fig. 10).

### Differential abundance

**Fig. 11:**
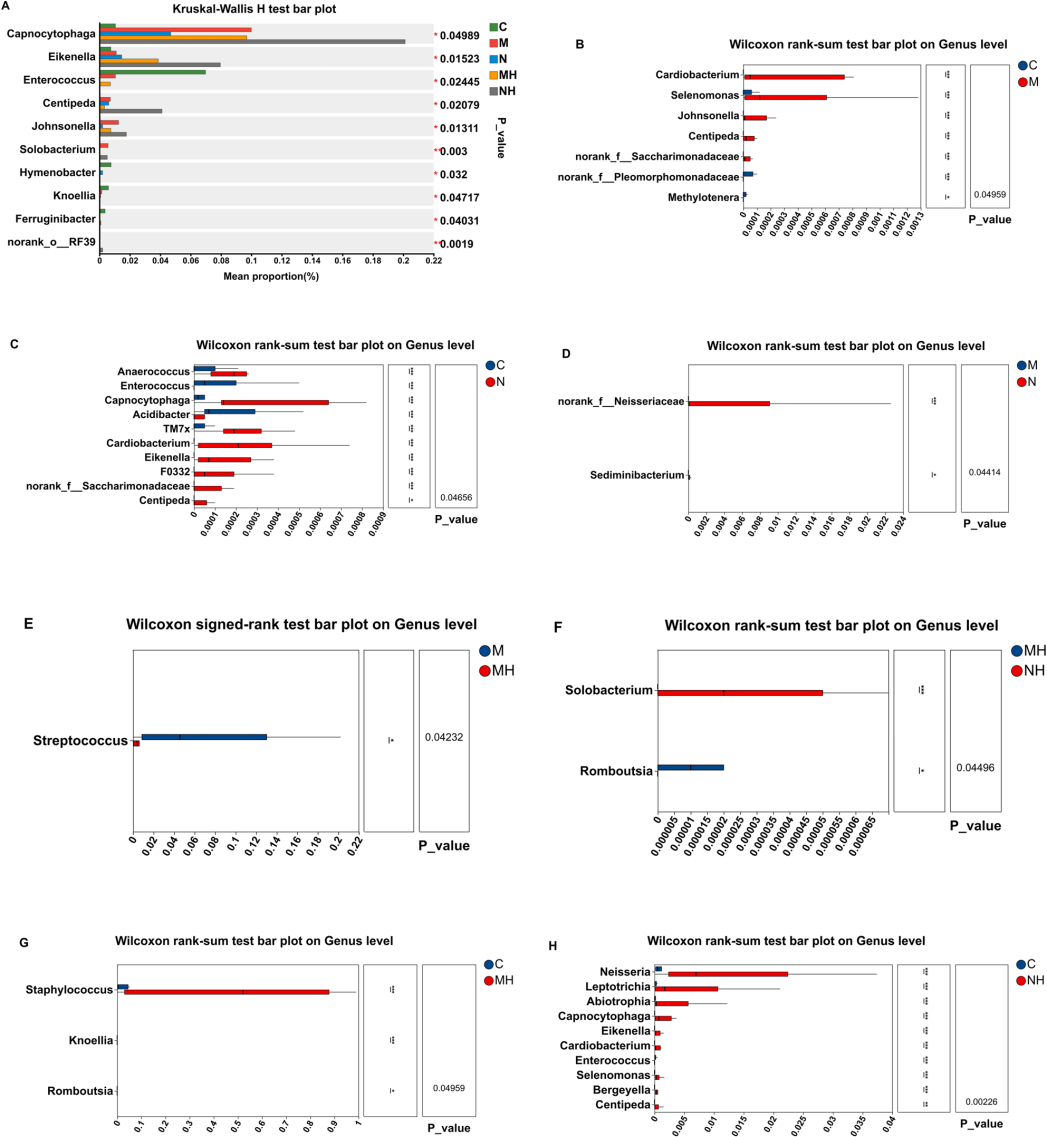
**A** Differential taxa among five groups. **B** Differential taxa between the control (C) and mouth-breathing (M) groups. **C** Differential taxa between the control (C) and nasal-breathing (N) groups. **D** Differential taxa between the mouth-breathing (M) and nasal-breathing (N) groups. **E** Differential taxa between the mouth-breathing before RME (M) and after RME (MH) groups. **F** Differential taxa between the mouth-breathing after RME (MH) and nasal-breathing after RME (NH) groups. **G** Differential taxa between the control (C) and mouth-breathing after RME (MH) groups. **H** Differential taxa between the control (C) and nasal-breathing after RME (NH) groups. The left bar chart shows the relative abundances of significantly different genera between the two groups, while the right panel indicates statistical significance and P-values (*P < 0.05, **P < 0.01)

Kruskal–Wallis testing identified several taxa with significant group-wise differences, including *Capnocytophaga, Eikenella, Enterococcus, Centipeda, Johnsonella,* and *Knoellia* (Fig. 11A). Pairwise comparisons revealed additional differences:

Compared with controls, mouth-breathing children showed higher abundances of *Cardiobacterium, Selenomonas, Johnsonella,* and *Centipeda*, while *Methylotenera* was enriched in controls (Fig. 11B).

In nasal-breathing children, *Anaerococcus, Capnocytophaga, Cardiobacterium, Eikenella,* and *Centipeda* were more abundant, whereas *Acidibacter* and *Enterococcus* were enriched in controls (Fig. 11C).

Pre-treatment comparisons showed higher *Sediminibacterium* and *unclassified Neisseriaceae* in nasal-breathing children (Fig. 11D).

Following RME, *Streptococcus* decreased significantly in mouth-breathing children (Fig. 11E), while no significant change was detected in nasal-breathing children.

Post-treatment, *Solobacterium* was enriched in nasal-breathing children, whereas *Romboutsia* was enriched in mouth-breathing children (Fig. 11F).

Compared with controls, post-RME mouth-breathing children had higher *Staphylococcus* and *Romboutsia,* while *Eikenella* was reduced (Fig. 11G).

Compared with controls, post-RME nasal-breathing children had higher abundances of *Neisseria, Leptotrichia, Abiotrophia, Capnocytophaga, Cardiobacterium, Eikenella, Selenomonas, Bergeyella,* and *Centipeda,* while *Enterococcus* was enriched in controls (Fig. 11H).

## Discussion

### Taxonomic annotation and clustering

The nasal cavity is a key component of the human microbiome and serves as the first barrier of the respiratory tract. Consistent with previous reports, our sequencing results identified a wide range of phyla and genera, but the distribution was dominated by a few abundant taxa, indicating unevenness of community structure. The shared presence of core taxa such as *Staphylococcus, Moraxella, Corynebacterium,* and *Dolosigranulum* in both mouth-breathing and control children suggests that a stable core nasal microbiome exists regardless of disease status. However, variations in relative abundance rather than taxonomic richness may contribute to differences in microbial community composition across groups.

### Alpha diversity

Alpha diversity indices (Chao1, ACE, Shannon, Simpson) indicated high richness and diversity across all samples with sufficient sequencing depth. Importantly, no significant differences in alpha diversity were observed between mouth-breathing (MTD) children and healthy controls, nor before versus after RME treatment. This finding aligns with previous studies reporting comparable nasal microbial diversity between patients and controls in chronic rhinosinusitis and other airway diseases [22]. The lack of diversity changes may be explained by the high homology of core nasal taxa across children [23]. Instead, alterations appear to occur at the level of taxonomic composition. Furthermore, heterogeneity in the etiology of mouth breathing (e.g., adenoid hypertrophy, allergic rhinitis, septal deviation) may cause divergent microbial shifts across individuals, thus obscuring group-wide statistical differences [24].

### Community composition

At the phylum level, Firmicutes, Proteobacteria, and Actinobacteria were consistently dominant, although RME was associated with distinct compositional shifts across subgroups. In mouth-breathing children, Firmicutes and Actinobacteria increased while Proteobacteria decreased, whereas in nasal-breathing children, Actinobacteria increased slightly with a concomitant reduction in Firmicutes and Proteobacteria. At the genus level, *Staphylococcus* and *Corynebacterium* increased after RME, particularly in the mouth-breathing subgroup, whereas *Moraxella*, *Dolosigranulum*, and *Haemophilus* decreased. These compositional shifts may reflect changes in the nasal microenvironment after RME. Prior studies have suggested that RME may be associated with structural and airflow-related changes in the upper airway [25–27]. However, airflow, mucociliary clearance, and host inflammatory responses were not directly measured in the present study, and mechanistic interpretations should therefore be considered speculative.

### Differential abundance

Differential abundance analysis identified several taxa that differed across groups. Compared with controls, mouth-breathing children showed higher relative abundance of *Cardiobacterium*, *Selenomonas*, *Johnsonella*, and *Centipeda*, whereas controls were enriched in *Methylotenera*. These differences may be related to variations in the local nasal microenvironment associated with breathing pattern; however, oxygenation status, mucus properties, and mucosal function were not directly assessed in this study [28–30].

In nasal-breathing children with MTD, taxa including *Anaerococcus*, *Capnocytophaga*, *Cardiobacterium*, *Eikenella*, and *Centipeda* were enriched compared with controls. Conversely, *Enterococcus* was more abundant in healthy children. Although these patterns may reflect differences in airway structure and local microenvironment, the underlying mechanisms remain uncertain and should be interpreted cautiously [30].

Longitudinally, RME was associated with a significant decrease in *Streptococcus* in mouth-breathing children, whereas no significant change was detected in nasal-breathing children. Given prior reports linking *Streptococcus* (including *S. pneumoniae*) to adenoid hypertrophy, allergic rhinitis, and airway inflammation [28, 31], this finding may be of potential clinical interest. However, inflammatory markers and mucosal outcomes were not measured in the present study, and the clinical significance of this compositional shift remains to be clarified.

### CBCT-based structural characterization of potential confounders

To further characterize potential structural confounders related to breathing pattern, we performed a descriptive CBCT-based review of the MTD subgroups (Supplementary Table S2). Adenoid-related hypertrophy, inferior turbinate hypertrophy, and nasal septal deviation were identified in both mouth-breathing and nasal-breathing children, although the distribution and apparent severity varied across individuals. These observations support the heterogeneity of airway-related structural features in children with MTD and suggest that visible structural findings on CBCT alone may be insufficient to fully explain breathing-pattern classification in this cohort. This descriptive review therefore complements our subgrouping strategy and helps contextualize the microbiota findings, while also indicating that non-imaging etiologies (e.g., allergic rhinitis) and functional factors may contribute to residual heterogeneity.

### Clinical implications

Taken together, these findings indicate that while overall nasal microbial diversity remained relatively stable, RME was associated with compositional shifts in the nasal microbiota of mouth-breathing children. In particular, post-RME changes included lower relative abundance of *Streptococcus*, *Moraxella*, and *Haemophilus*, and higher relative abundance of *Staphylococcus* and *Corynebacterium*. These observations may suggest changes toward a control-like compositional pattern; however, they should not be interpreted as evidence of restored microbial homeostasis or improved airway function, as functional, inflammatory, and standardized objective post-treatment breathing-status assessments were not performed in this study. The persistence of certain oral-associated taxa (e.g., *Romboutsia*, *Johnsonella*) after RME may reflect ongoing oral–nasal microbial exchange and underscores the need for longer-term follow-up.

### Limitations

Several limitations should be considered when interpreting the present findings. First, as an observational prospective cohort study, this study cannot establish causality. Second, the subgroup sample size was small (n = 8 per subgroup), which may limit statistical power and the stability of subgroup-specific findings. Third, breathing-pattern subgrouping was based on a combination of clinical record review (including available documentation of “mouth breathing”/“chronic mouth breathing” and related symptom history), caregiver-reported breathing habit (including during sleep), and clinical observation (including chairside fogging mirror–based assessment to assist evaluation of oral airflow), rather than a standardized objective airflow-testing protocol; therefore, misclassification bias cannot be excluded. ENT-related history/diagnostic information was available in clinical records for subgroup characterization, but original ENT consultation documents were not uniformly available for all participants, and some ENT recommendations were caregiver-reported. Although children with prior adenotonsillar surgery were excluded to reduce potential microbiota-related confounding, residual confounding related to mouth-breathing etiology remains possible. In addition, a descriptive CBCT-based review of the MTD subgroups identified structural findings such as adenoid-related hypertrophy, inferior turbinate hypertrophy, and nasal septal deviation in both mouth-breathing and nasal-breathing children (Supplementary Table S2), indicating heterogeneity of airway-related structural features within this cohort. However, non-imaging etiologies of mouth breathing (e.g., allergic rhinitis) could not be determined from CBCT and were not uniformly documented using standardized ENT records. MTD diagnosis and indication for RME were based on routine orthodontic clinical evaluation supported by CBCT-based transverse assessment (using the Penn analysis framework), dental casts, and occlusal photographs; however, the present study was designed to evaluate microbiota compositional differences rather than to perform a detailed imaging-based orthodontic analysis. Fifth, samples were collected at approximately one month after completion of active expansion, and thus the findings reflect short-term compositional changes rather than long-term persistence. At the post-RME follow-up visit (approximately one month after completion of active expansion, during retention), we performed a symptom-focused clinical reassessment using the same chairside approach as at baseline (including caregiver-reported nighttime mouth breathing/snoring and clinical observation with a fogging mirror–based assessment of oral airflow). In routine clinical observation, mouth-breathing symptoms appeared improved in most children. However, post-treatment breathing status was not reassessed using standardized otolaryngological objective methods; therefore, the relationship between post-RME microbiota compositional shifts and changes in breathing pattern should be interpreted with caution. Finally, 16S rRNA profiling provides compositional information only, and inflammatory markers, mucosal outcomes, and functional microbial validation were not assessed.

### Future directions

Future studies with larger cohorts, standardized post-treatment otolaryngological reassessment, and multi-omics approaches (e.g., metagenomics or metabolomics) are warranted to clarify the mechanistic links among RME, airway status, host responses, and microbial ecology.

## Conclusions

In this prospective cohort study, RME did not substantially alter overall nasal microbial alpha diversity but was associated with short-term compositional shifts in mouth-breathing children with MTD. These shifts included reduced relative abundance of *Streptococcus* and increased relative abundance of *Staphylococcus* and *Corynebacterium* after RME.

These findings should be interpreted as exploratory associations rather than evidence of causal or functional treatment effects. They suggest that RME may be associated with short-term changes in upper airway microbial composition beyond correction of maxillary constriction, but the clinical significance of these changes remains uncertain. Given the limited sample size and short follow-up, larger longitudinal studies incorporating standardized breathing-pattern reassessment and functional/inflammatory outcome measures are needed.

## Supplementary Information

Supplementary Material 1: Supplementary Table S1 (diagnostic transparency summary for MTD diagnosis and breathing-pattern subgrouping), Supplementary Table S2 (CBCT-based structural findings related to potential upper airway confounders in the MTD subgroups), Supplementary Figures S1–S5 (representative DNA electrophoresis results and full uncropped PCR gel images), and Supplementary Figure S6 (participant flow diagram).

## Data Availability

The raw 16S rRNA gene sequencing data generated in this study have been deposited in the NCBI Sequence Read Archive (SRA) under BioProject accession PRJNA1221664 (https://www.ncbi.nlm.nih.gov/bioproject/PRJNA1221664) and are publicly available. Other data supporting the findings of this study are included within the article and its supplementary information files.

## Abbreviations

MTD: Maxillary Transverse Deficiency
RME: Rapid Maxillary Expansion
PCR: Polymerase Chain Reaction
OTUs: Operational Taxonomic Units
PCA: Principal Component Analysis
PCoA: Principal Coordinates Analysis
ACE: Abundance-based Coverage Estimator
