# Supplementary material for: Impact of rapid maxillary expansion on nasal microbiota in mouth-breathing children: a prospective cohort study
Source: BMC Oral Health. 2026 Mar 28;26:798. doi: 10.1186/s12903-026-08148-2 (PMC13151359; doi:10.1186/s12903-026-08148-2)
Supplement: Supplementary file 1 — Supplementary Material 1: Supplementary Table S1 (diagnostic transparency summary for MTD diagnosis and breathing-pattern subgrouping), Supplementary Table S2 (CBCT-based structural findings related to potential upper airway confounders in the MTD subgroups), Supplementary Figures S1–S5 (representative DNA electrophoresis results and full uncropped PCR gel images), and Supplementary Figure S6 (participant flow diagram). [file 12903_2026_8148_MOESM1_ESM.docx]

## ****Supplementary Materials****

This supplementary file includes additional methodological details and supporting materials for the manuscript entitled:

**“Impact of rapid maxillary expansion on nasal microbiota in mouth-breathing children: a prospective cohort study”**

### ****Contents****

1. **Supplementary Methods**

- Orthodontic and breathing-pattern assessment

1. **Supplementary Table S1**

- Diagnostic transparency summary for MTD diagnosis and breathing-pattern subgrouping

1. **Supplementary Table S2**

- CBCT-based structural findings related to potential upper airway confounders in the MTD subgroups

1. **Supplementary Figure S1–S2**

- Representative DNA electrophoresis results

1. **Supplementary Figure S3–S5**

- Full uncropped PCR gel images

1. **Supplementary Figure S6**

- Participant flow diagram (screening, exclusions, group allocation, sampling time points, and analyzed samples)

**Abbreviations:** MTD, maxillary transverse deficiency; RME, rapid maxillary expansion.

# Supplementary Methods: Orthodontic and breathing-pattern assessment

**Orthodontic assessment for MTD diagnosis and RME indication**
Children in the MTD group were identified through routine orthodontic clinical evaluation and treatment planning for rapid maxillary expansion (RME). Clinical examination could reveal suggestive findings such as apparent transverse insufficiency, maxillary arch narrowing, or dental crowding; however, these findings alone were not treated as definitive diagnostic criteria for MTD. Posterior crossbite, when present, was considered a strong clinical indicator of MTD and a likely indication for expansion. All children in the MTD group underwent CBCT as part of routine orthodontic records, and CBCT-based transverse assessment, including maxillary and mandibular basal bone width evaluation using the Penn analysis framework, served as the principal imaging support for diagnosis and treatment planning. Additional orthodontic records, including routine clinical examination, dental casts, and occlusal photographs, were also reviewed. The orthodontic treatment plan (including indication for RME) was formulated by the same senior orthodontist to improve diagnostic and treatment-planning consistency.

**Breathing-pattern subgrouping (mouth-breathing vs nasal-breathing)**

Breathing-pattern subgrouping was performed before treatment using a combination of (1) medical record review, (2) caregiver-reported breathing habit (including during sleep), and (3) clinical observation.

For the mouth-breathing subgroup, supporting information included one or more of the following:

- medical record documentation of “mouth breathing” or “chronic mouth breathing”;
- history records suggestive of chronic mouth breathing (e.g., frequent/long-term snoring, nasal obstruction symptoms, nighttime mouth opening);
- caregiver-reported nighttime mouth breathing and/or snoring;
- clinical signs suggestive of mouth breathing (e.g., habitual open-mouth posture, lip eversion/lip incompetence, mouth-breathing facial features), with chairside clinical observation including a fogging mirror–based assessment to assist evaluation of oral airflow through the mouth.

Children in the nasal-breathing subgroup had no clinically observed mouth-breathing symptoms, and caregivers reported no mouth breathing during sleep.

ENT-related history/diagnostic information (e.g., rhinitis/sinusitis/adenoid-related history or prior ENT visits) was reviewed when documented in the available records. ENT consultation recommendations were sometimes reported by caregivers; however, original ENT consultation documents were not uniformly available for all participants.

Children with prior adenoidectomy or tonsillectomy were excluded to reduce potential microbiota-related confounding, as adenotonsillar surgery may influence nasal microbial community structure.

To further characterize potential structural confounders related to breathing pattern, CBCT images of the MTD subgroups were additionally reviewed (with supervising clinician confirmation) for the presence/severity of adenoid-related hypertrophy, inferior turbinate hypertrophy, and nasal septal deviation, and summarized descriptively in Supplementary Table S2.

At the post-RME follow-up visit, a symptom-focused reassessment using the same chairside approach (caregiver report and fogging mirror–based oral airflow observation) was performed as part of routine follow-up, but standardized objective ENT reassessment was not conducted.

Breathing-pattern subgrouping was based on clinical records, caregiver report, and clinical observation rather than a standardized objective airflow-testing protocol. Therefore, potential misclassification bias (including mixed or dynamic breathing patterns) cannot be excluded.

# Supplementary Table S1. Diagnostic transparency summary for MTD diagnosis and breathing-pattern subgrouping

| **Domain** | **Item** | **What was used in this study** | **Notes / limitations** |
| --- | --- | --- | --- |
| Orthodontic assessment | Purpose | To identify maxillary transverse deficiency (MTD) and determine the indication for rapid maxillary expansion (RME) | — |
| Orthodontic assessment | Key clinical findings considered | Posterior crossbite on clinical examination (when present) | Not all children with MTD presented with posterior crossbite |
| Orthodontic assessment | CBCT-based support | CBCT-based transverse assessment using the Penn analysis framework, including maxillary and mandibular basal bone width assessment | Used to support MTD diagnosis and treatment planning |
| Orthodontic assessment | Additional orthodontic records | Routine orthodontic clinical examination; CBCT (all MTD participants); dental casts; occlusal photographs | Used as part of routine orthodontic records and treatment planning |
| Orthodontic assessment | Treatment planning | The indication for RME and treatment plan were determined by the same senior orthodontist | Intended to improve diagnostic and treatment-planning consistency |
| Breathing-pattern subgrouping | Purpose | To classify children into mouth-breathing and nasal-breathing subgroups before treatment | Subgrouping was performed before RME |
| Breathing-pattern subgrouping | Data sources | Medical record review, caregiver-reported breathing habit (including during sleep), and clinical observation | Not based on a standardized objective airflow-testing protocol |
| Mouth-breathing subgroup | Supporting evidence | Medical record documentation of “mouth breathing” / “chronic mouth breathing”; symptom history suggestive of chronic mouth breathing (e.g., frequent/long-term snoring, nasal obstruction symptoms, nighttime mouth opening); caregiver-reported nighttime mouth breathing and/or snoring; clinical signs including habitual open-mouth posture, lip eversion/lip incompetence, mouth-breathing facial features, and fogging mirror–based oral airflow assessment | ENT-related history/diagnostic information was reviewed when available in clinical records |
| Nasal-breathing subgroup | Supporting evidence | No clinically observed mouth-breathing symptoms and no caregiver-reported mouth breathing during sleep | Mixed or dynamic breathing patterns cannot be fully excluded |
| Control group | Inclusion characteristics | Normal maxillary transverse development, no anterior or posterior crossbite, no maxillary arch constriction, and no orthodontic treatment need | Healthy age-matched children |
| Exclusion criteria | Shared exclusions | Recent use of antibiotics, probiotics, or nasal sprays; prior adenoidectomy, tonsillectomy, or other nasal/nasopharyngeal surgery; prior airway-related treatment (e.g., CPAP, oral appliances, UPPP); syndromes, chronic systemic disease, craniofacial deformities, complete nasal obstruction, septal perforation, or acute oral/nasal/pharyngeal disease | Prior adenotonsillar surgery was excluded because it may influence nasal microbial community structure |

**Abbreviations:** MTD, maxillary transverse deficiency; RME, rapid maxillary expansion; CBCT, cone-beam computed tomography; CPAP, continuous positive airway pressure; UPPP, uvulopalatopharyngoplasty.

# Supplementary Table S2. CBCT-based structural findings related to potential upper airway confounders in the MTD subgroups

MTD subgroup size: Mouth-breathing MTD (n = 8); Nasal-breathing MTD (n = 8)

| **CBCT-based structural finding** | **Mouth-breathing MTD (n = 8)** | **Nasal-breathing MTD (n = 8)** |
| --- | --- | --- |
| Adenoid-related hypertrophy (any) | 1 (moderate) | 1 (mild) |
| Inferior turbinate hypertrophy (any) | 3 (2 marked, 1 mild) | 3 (1 marked, 2 mild) |
| Nasal septal deviation (any) | 4 (2 marked, 2 mild) | 4 (2 marked, 2 mild) |

**Abbreviations:** MTD, maxillary transverse deficiency; CBCT, cone-beam computed tomography.
CBCT findings were recorded as structural features for confounder characterization in the MTD subgroups based on image review and clinician verification. These findings were summarized descriptively and were not intended to replace formal otolaryngological diagnosis. Non-imaging etiologies of mouth breathing (e.g., allergic rhinitis) cannot be determined from CBCT.

# Supplementary Figures S1–S5


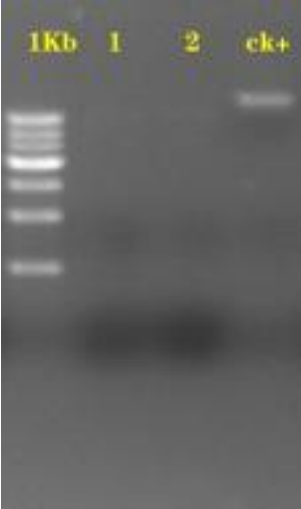


**Supplementary Figure S1.** DNA electrophoresis of pilot samples (n = 2). Genomic DNA was extracted from two nasal swab samples in a pilot test and analyzed by 1% agarose gel electrophoresis. Lane 1Kb: 1 kb DNA ladder (bands at 1000–10,000 bp); lanes 1–2: representative DNA samples from the pilot cohort; lane ck+: positive control. Clear high-molecular-weight DNA bands are visible.


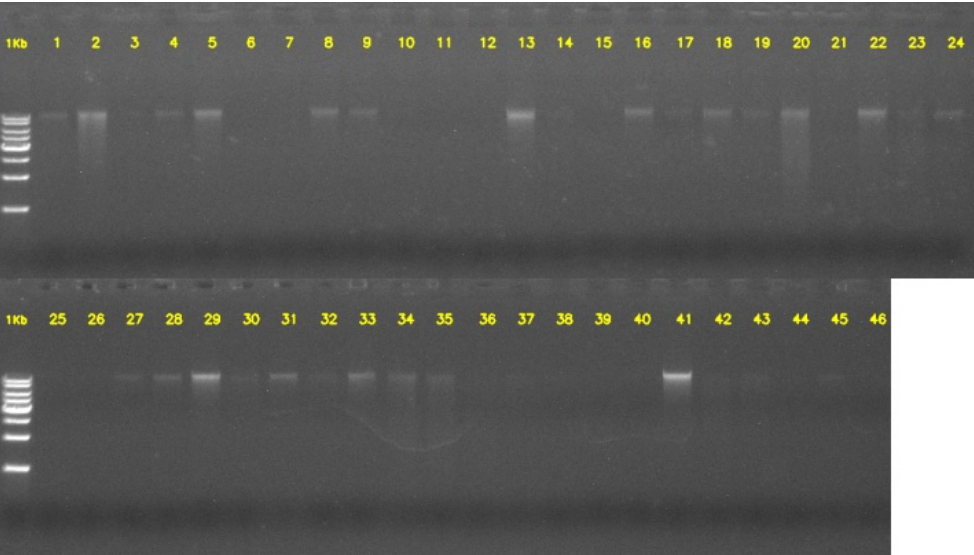


**Supplementary Figure S2.** DNA electrophoresis of the main cohort (n = 46). Genomic DNA was extracted from nasal swabs in the main experiment and verified on 1% agarose gels. Lane 1Kb: 1 kb DNA ladder; lanes 1–46: DNA samples from the main cohort. Most lanes show intact high-molecular-weight DNA bands, confirming sufficient quality for downstream PCR amplification and sequencing.


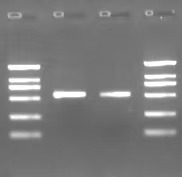


**Supplementary Figure S3.** PCR amplification gel electrophoresis of pilot samples (n = 2). Two nasal swab DNA samples were tested to validate PCR amplification of the 16S rRNA gene V3–V4 region. Lane M: DL2000 DNA marker (bands at 2000, 1000, 750, 500, 250, and 100 bp); lanes 1–2: pilot PCR amplicons; lane CK: negative control. Clear target bands are observed at ~750 bp.


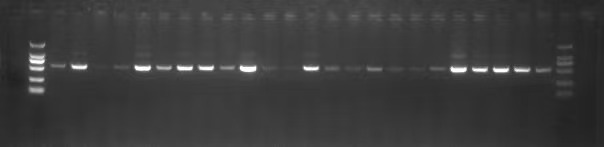


**Supplementary Figure S4.** PCR amplification gel electrophoresis of the main cohort (n = 23, batch 1). Lane M: DL2000 DNA marker; lanes 3–25: representative PCR amplicons of the 16S rRNA gene V3–V4 region from the main cohort; lane CK: negative control. Specific target bands are observed at ~750 bp.


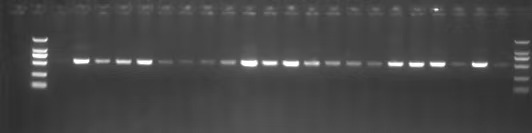


**Supplementary Figure S5.** PCR amplification gel electrophoresis of the main cohort (n = 23, batch 2). Lane M: DL2000 DNA marker; lanes 26–46: PCR amplicons of the 16S rRNA gene V3–V4 region from the main cohort; lane CK: negative control. Distinct bands of the expected size (~750 bp) confirm successful amplification.

# Supplementary Figure S6. Participant flow diagram


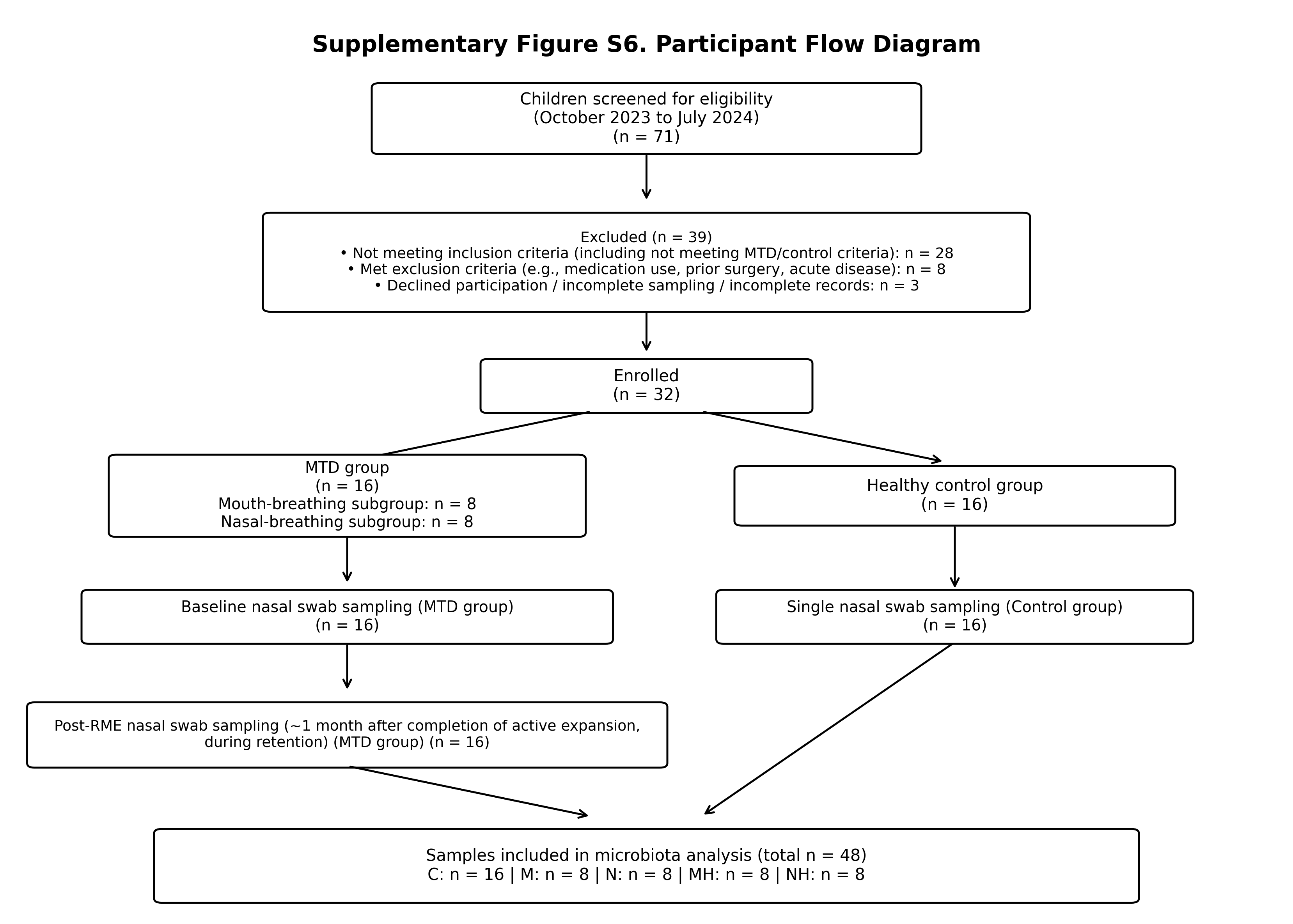


Participant flow diagram showing screening, exclusions (with reasons), enrollment, group allocation, sampling time points, and samples included in the microbiota analysis. A total of 71 children were screened, 39 were excluded, and 32 were enrolled (16 with maxillary transverse deficiency [MTD] and 16 healthy controls). The MTD group included 8 mouth-breathing and 8 nasal-breathing children. Nasal swabs were collected at baseline and approximately one month after completion of active expansion (during retention) in the MTD group, and once in the control group, yielding 48 samples for analysis.
